# Nutrition and Rheumatoid Arthritis in the ‘Omics’ Era

**DOI:** 10.3390/nu13030763

**Published:** 2021-02-26

**Authors:** Manuela Cassotta, Tamara Y. Forbes-Hernandez, Danila Cianciosi, Maria Elexpuru Zabaleta, Sandra Sumalla Cano, Irma Dominguez, Beatriz Bullon, Lucia Regolo, Josè Miguel Alvarez-Suarez, Francesca Giampieri, Maurizio Battino

**Affiliations:** 1 Research Group on Foods, Nutritional Biochemistry and Health, Universidad Europea del Atlántico, 39011 Santander, Spain; manuela.cassotta@gmail.com (M.C.); maria.elexpuru@uneatlantico.es (M.E.Z.); sandra.sumalla@uneatlantico.es (S.S.C.); irma.dominguez@uneatlantico.es (I.D.); 2 Nutrition and Food Science Group, Department of Analytical and Food Chemistry, CITACA, CACTI, University of Vigo, 36310 Vigo, Spain; tforbes@uvigo.es; 3 Department of Clinical Sciences, Faculty of Medicine, Polytechnic University of Marche, 60131 Ancona, Italy; d.cianciosi@pm.univpm.it (D.C.); luciaregolo@gmail.com (L.R.); 4 Department of Periodontology, Dental School, University of Sevilla, 41004 Sevilla, Spain; beatrizbullon@hotmail.com; 5 AgroScience & Food Research Group, Universidad de Las Américas, Quito 170125, Ecuador; jose.alvarez@udla.edu.ec; 6 King Fahd Medical Research Center, King Abdulaziz University, Jedda 21589, Saudi Arabia; 7 Department of Biochemistry, Faculty of Sciences, King Abdulaziz University, Jeddah 21589, Saudi Arabia; 8 International Research Center for Food Nutrition and Safety, Jiangsu University, Zhenjiang 212013, China

**Keywords:** rheumatoid arthritis, diet, nutrigenomics, proteomics, metabolomics, microbiome, microbiomics

## Abstract

Modern high-throughput ‘omics’ science tools (including genomics, transcriptomics, proteomics, metabolomics and microbiomics) are currently being applied to nutritional sciences to unravel the fundamental processes of health effects ascribed to particular nutrients in humans and to contribute to more precise nutritional advice. Diet and food components are key environmental factors that interact with the genome, transcriptome, proteome, metabolome and the microbiota, and this life-long interplay defines health and diseases state of the individual. Rheumatoid arthritis (RA) is a chronic autoimmune disease featured by a systemic immune-inflammatory response, in genetically susceptible individuals exposed to environmental triggers, including diet. In recent years increasing evidences suggested that nutritional factors and gut microbiome have a central role in RA risk and progression. The aim of this review is to summarize the main and most recent applications of ‘omics’ technologies in human nutrition and in RA research, examining the possible influences of some nutrients and nutritional patterns on RA pathogenesis, following a nutrigenomics approach. The opportunities and challenges of novel ‘omics technologies’ in the exploration of new avenues in RA and nutritional research to prevent and manage RA will be also discussed.

## 1. Introduction

Recent advances in high-throughput/high-content techniques have led to a new framework in biomedical research, the so-called ‘Omics era’, which combines the opportunity to gather great amounts of data and details at the molecular level together with the evolution of new computational models and statistical tools that are able to analyze and filter such data. Then, progresses in next generation sequencing (NGS), high-throughput platforms, mass-spectrometry and bioinformatic equipment, enabled the synchronous extensive study of thousands of genes (genomics), epigenetic factors (epigenomics), RNA (transcriptomics), metabolites (metabolomics) proteins (proteomics), and human-microbiota (microbiomics), with the potential of combining diverse categories of ‘omics’ data (‘multi-omics’ or ‘system biology’). Those novel ‘omics’ approaches and techniques have revolutionized the study of complex human diseases, providing an unparalleled genome-wide view of genetic variation, gene expression, interaction with microbiota, and environmentally responsive epigenetic changes [1,2,3]. Moreover, nutritional research has shifted from traditional physiology and epidemiology to molecular genetics and biology. Applications of the above mentioned ‘omics’ facilitated molecular nutrition understanding. Pursuing this course, nutrigenomics has developed as a pluridisciplinary research field in nutrition-science that intends to clarify how nutrition can affect human health. While the first meaning of nutrigenomics concerned only with studies on nutrients or bioactive food compounds influencing gene expression of a person, currently, this definition has been extended and, in recent times, nutrigenomics refers to the employment of transcriptomics, genomics, proteomics, metabolomics and epigenomics to find out and elucidate the existing mutual relations between genes and nutrients at a molecular level, also encompassing nutrigenetic studies exploring the relationships between genetic variants and diet in modulating disease risk [4]. In this review, we use the term “nutrigenomics” in its broadest definition. Rheumatoid arthritis (RA) is a persistent inflammatory autoimmune disorder which affects roughly 1% of the global population. It is characterized by extensive synovitis, systemic inflammation and various degrees of cartilage and bone erosion. Multiple genetic and environmental factors, including modifiable lifestyle factors such as cigarette smoking and dietary habits have been linked with an increased risk for RA. Cumulative evidences suggested that nutrition has a central role in RA risk and progression [5,6]. It is already widely-known that bioactive food components can interact with genes impacting transcription factors, protein expression, metabolite production and microbiota. On the other hand, the genetic makeup of the single person can delineate the nutritional state, metabolic responses, and predisposition to diet-associated health disorders. Research in this field can help us to comprehend why some people respond differently from others to the same foods, beverages and supplements. Recent evidence has shown that the host microbiota, and especially the intestinal microbiota, play a central role in the onset and progression of several diseases, including RA [7,8]. Accelerated progress of high-throughput molecular technologies has allowed to conduct meticulous studies of the microbiota in humans, exploring the biological signatures that are connected with definite illnesses, environmental conditions, or pharmacological/nutritional interventions [9,10,11,12,13]. Nutrition is the main modulator of bacterial composition and abundance in the gastrointestinal tract, suggesting the opportunities for therapeutic nutritional approaches to manipulate microbiota composition and diversity [14,15]. Nutrigenomics and nutritional microbiomics approaches have received increasing attention and are currently being used to study respectively the mutual interactions between food and genes and between diet and microbiota in several diseases including cancer [16,17], cardiovascular [18,19], metabolic [20], and autoimmune diseases [21,22,23,24] in order to enable their better understanding, prevention and treatment through optimization of individuals’ dietary intakes.

Despite important research advancements and clinical improvements, RA still represents a public health challenge, in terms of both epidemiological and economic burden: many patients still experience premature work disability, co-morbidities, and important adverse effects caused by medications. Although with availability of several disease-modifying antirheumatic drugs and biologic therapies the outcomes for patients with RA have significantly improved, RA remains a long-standing condition for which there is currently no effective cure [25]. Extensive use of the ever expanding, novel ‘omics’ technologies will facilitate both the understanding of RA pathogenesis and the identification and modification of nutritional-related risk factors, allowing to establish a prevention strategy for RA in susceptible population as well as to complement the present treatment strategies for a better disease management. The combined-use of several ‘omics’ technologies will also enable the discovery of novel biomarkers related with specific food or dietary intake, greatly facilitating human nutritional studies. After presenting an overview of the most recent genomics, transcriptomics, proteomics, metabolomics, epigenomics, and microbiomics approaches and their implementation within human nutrition research, in this paper we review the role of ‘omics’ technologies in elucidating the pathogenesis of RA and the possible influence of some nutrients, food bioactive compounds, nutritional patterns and microbiota on RA pathogenesis.

The aim of this review is to present the main novel ‘omics’ approaches and their current and possible future application in RA and nutrition research, with the ultimate goal to promote a human-relevant nutrigenomics approach to RA for disease prevention and better disease management. The opportunities and challenges of novel ‘omics technologies’ will be also discussed.

Literature search was conducted using PubMed (between August–November 2020) and different combinations of search terms and Boolean operators (see Supplementary Table S1). Only human-based studies (e.g., observational and interventional studies in RA and inflammation) were reviewed.

## 2. Applications of ‘Omics’ Approaches and Technologies within Nutritional Research

A crucial goal of nutritional research is to establish the role of nutrition in metabolic control and to boost health. Nutrition and health correlations have been classically explained in terms of energetic and structural necessity of the body, as assured by nutrients. Nevertheless, foods also include several secondary biologically active non-nutrient compounds which can also help in the prevention and even in the treatment of various chronic diseases. In fact many epidemiological studies have shown a correlation between nutrition and the incidence of different disorders, in particular type 2 diabetes [26,27], cardiovascular diseases [28,29,30], cancer [31,32,33,34], neurodegenerative disorders [35,36], and RA [5,37,38]. However, the understanding of the exact components and the mechanisms underlying their supposed beneficial or detrimental effects is still insufficient. Newly introduced modern high throughput ‘omics’ approaches are greatly contributing to the elucidation of the connections between dietary exposure and health at the molecular level [39,40]. Genomics, transcriptomics, proteomics, and metabolomics are four main platforms of comprehensive ‘omics’ approach in nutritional science. They respectively investigate the whole set of DNA, RNA, proteins and metabolites in a cell, tissue or entire organism. The start of the 21st century was featured by prompt progress in high-throughput technologies, high-content- and single-cell approaches, mass spectrometry, bioinformatics, and computing capacities. These tools are widely used in an effort to identify molecular events implicated in the health effects of nutritional components or in diet-related diseases [41]. Nutrigenetics is the science that detects and describes gene variants linked with differential response to nutrients and relating this variation to different disease states. Next generation sequencing (NGS), also known as ‘second-generation sequencing’, makes it possible to read the code of great amounts of small fragments of DNA or RNA in parallel, enabling more rapid sequencing with higher throughput at dropping costs. Genome-wide association studies together with NGS have contributed to identify novel genomic variants (for e.g., genetic polymorphisms) with the aim of understanding complex disease pathobiology as well as examining the influences of dietary exposure and genetic variants in humans. The integration of genetic polymorphisms into nutritional epidemiological studies has allowed to tackle several limitations intrinsic in such studies, such as genetic variability affecting either the absorption, biotransformation, metabolism, distribution or elimination of a nutrient or bioactive food compound [42,43]. One example of how nutrigenomics and NGS technologies has been employed to elucidate the involvement of precise dietary factors results from an investigation on coffee and heart disease [44]. Numerous studies had examined this relationship and deduced that coffee either decreases risk, has no impact, or increases risk [45]. Even tough coffee is a complex mixture consisting of a large amount of bioactive compounds, it is an important source of caffeine and it has always been recognized that caffeine might be particularly deleterious for the cardiovascular system. Caffeinated-coffee was found to increase the risk of heart infarction among persons who carry a gene variant that makes them ‘slow’ caffeine metabolizers, but do not affect individuals who are ‘fast’ caffeine metabolizers [46]. NGS and omics-based readouts applied to nutrigenetics, will give crucially significant information that will support clinicians in ensuring the optimal diet for a particular individual, namely personalized nutrition.

Transcriptomics studies have become routine, thanks to technologies such as real-time-PCR and robust microarrays. Moreover, RNA sequencing has developed as a robust alternative for transcriptome investigation as it is covering wider spectrum of RNAs ensuring more useful information [47,48]. Transcriptomics analysis provides opportunities to investigate the transcriptome at a given nutritional condition, affording a comprehensive view of intracellular RNA expression [49]. For example, transcription profiling has been widely applied to assess the possible effects of anthocyanins, pigments naturally occurring in many comestible vegetables and fruits, on obesity associated gene expression in human adipocytes [50] and also for exploring the potentialities of gene expression profiling in blood to investigate the influences of nutritional exposure in human intervention studies [51]. Nutri-miromics explores the impact of diet on gene expression as a result of epigenetic processes related to microRNAs (miRNAs), which may influence the risk for the development of chronic diseases, including RA [52,53]. MiRNAs are small non-coding endogenous RNA molecules that functions in post-transcriptional regulation of gene expression by causing mRNA degradation or translational suppression through binding to a target messenger RNA. They may be regulated by environmental and nutritional factors, mainly by single nutrients or bioactive food components [54], suggesting that diet manipulation may have the potential to serve as a therapeutic approach in controlling the risk of chronic diseases. It has been suggested that miRNAs not only are synthesized endogenously, but also might be acquired from diet [55]. Although such subject is still debated, it has been proposed that exogenous miRNAs may modulate serum miRNAs profiles possibly influencing biological processes [56]. Next-generation sequencing and omics-based analyses provides a powerful tool to identify dietary miRNAs as well as to understand the complex crosstalk between nutrition, miRNAs, gene targets, and human health and disease [57]. Extensive availability of NGS and ‘multi-omics’ technologies has allowed genome-wide clarification of the epigenomic makeup in dozens of cell types, throughout developmental times, in several species, including humans [58].

Nutritional epigenomics deals with assessing the influence of nutrition and bioactive food compounds on global epigenetic mechanisms that regulate gene activity and expression, for e.g., DNA methylation, histone modifications (histone methylation, acetylation, and phosphorylation), chromatin remodelling, and noncoding RNAs. Epigenetic mechanisms have been implicated in pathogenesis of several diseases, including autoimmune conditions [59,60,61,62,63]. Since nutrition is among the most significant environmental factors influencing the epigenetic profile, nutritional-epigenomics powered by novel technologies is emerging as a promising approach in nutritional research and for personalized nutrition [64]. In the past few years, new technologies that allow to sequence longer strands of nucleic acids by reading single DNA or RNA molecules have made progress and become more eminent [65]. These technologies, which can also be called ‘long-read sequencing’ or ‘third generation sequencing’, together with the development in bioinformatics equipment and single-cell sequencing methodods, will allow to decipher human genome, transcriptome, microbiome and epigenome to a greater depth [66,67,68,69,70]. These technologies are already being successful applied in nutritional sciences to investigate, for e.g., the effect of dietary interventions on human microbiome [71].

Metabolomics is referred to the study of the overall metabolites set or small molecules (metabolome) present in biological samples. Conventionally, Nuclear Magnetic Resonance (NMR), Proton Nuclear Magnetic Resonance (^1^HNMR) Spectroscopy, and Mass Spectrometry (MS) have represented the principal technologies employed in metabolomics studies. Progress in these tools, in particular Matrix-Assisted Laser Desorption Ionization Time Of Flight (MALDI-TOF), Secondary Ion Mass Spectrometry (SIMS), and Fourier transform ion cyclotron resonance MS, along with new approaches for compound identification [72] have opened numerous opportunities for quantitative, non-invasive analysis for metabolites in human-body fluids and tissues as well as changes thereon in response to diet. Nutritional metabolomics has emerged as high-performance and sensitive method for the identification and characterization of biochemical pathways [73]. Moreover, it has been used in several studies for assessing metabolite profiles as a result of specific dietary intake [74,75,76,77] and several metabolic perturbations were detected. To refine the assessment of a person’s food intake, and therefore elucidate suggested correlations between diet and disease, relevant and accurate dietary evaluation methods are crucial. Dietary biomarkers have arisen as a complementary tool to the conventional methods in nutrition studies, and in the few past years, metabolomics has established as an important approach for the identification of new dietary biomarkers [78]. Proteomics is the large-scale, high-throughput study of the expression, structure, function, modifications, and interactions of proteins, within a biofluid or tissue sample. A broad spectrum of different approaches and methods are being utilized for proteomic studies, particularly microarray-based tools, mass-spectrometry, nuclear-magnetic resonance, and the most modern single-cell and high-sensitivity protein analyses [79,80,81]. Nutritional proteomics or nutriproteomics harnesses proteomics technologies to identify molecular and cellular variations in protein expression and function on a comprehensive level as well as evaluating the interaction of proteins with food components. Food components may interact with endogenous proteins inducing post-translational modifications and modulating their original functions. The characterization of such modifications will allow a better understanding of the interplay between bioactive dietary components and diet-related diseases [82,83]. For e.g., nutriproteomics could help in elucidating the possible relationships between food antigens and autoimmune disorders [84]. Tsuda et al. (2015) have demonstrated that monoclonal autoantibodies derived from RA patients cross-react not only with various autoantigens but also with numerous food proteins. The authors proposed that such dietary proteins may trigger the generation of RA-specific autoantibodies to induce autoimmunity in at risk individuals [85]. Interestingly, it has been also hypothesized that chemical alterations of food proteins by different toxic agents in food may lead to immune reaction against altered food proteins that cross-react with tissue antigens, causing autoimmune reactions [84]. Understanding the possible link between specific food consumption and autoimmunity in humans may lead to prevention of autoimmune diseases through precise dietary advices in at risk individuals. From birth, humans interact and coevolve with trillions of microbes residing in most body surfaces and cavities, referred to as the human microbiota. Advances in ‘omics’ technologies and computational methods have driven the investigation of the microbiota’s contribution to human health and disease, led by massive efforts such as the Human Microbiome Project and the Europe-based MetaHit Consortium [86]. Microbiomics is an emerging rapidly-growing field in which all the microbes of a particular community (for e.g., gut microbiota) are analyzed together, harnessing ‘omics’ approaches and technologies, including metagenomics, metatranscriptomics, metaproteomics and metabolomics. These technologies, which investigate respectively the collective genome, transcriptome, proteome, and metabolome of microorganisms from a sample (e.g., human stools or saliva), are providing information concerning the structure and function of the entire microbial community as well as the identification and assessment of regulatory and metabolic machinery by which host and microbes interact among themselves to determine a healthy or diseased state in the human host [87]. Every single microbial genus in the gut comprises numerous species and strains that may harbour important dissimilarities in their genomes and functional characteristics and it has been documented that strain-level diversity may result in inconsistencies in genus and species associations with dietary interventions, health or disease [88,89]. Our data frequently are based on a genus- or species-level taxonomic allocations that, even if helpful, may not be adequate for an exhaustive comprehension of the complex relations between the gut microbiome, diet and human health. As sequencing technologies continue to evolve, novel strain-level understandings can be achieved in the study of the relationships between gut microbiota, diet and human health [90]. Microbiome composition has been linked to disease also by way of modulation of diet-derived specific metabolites and signaling pathways [91,92]. Thus, nutritional microbiomics and metabolomics studies hold promise for the discovery of pathways linked to disease processes. Nutritional microbiomics is a promising approach to investigate the interaction between diet and gut microbiota and the potential of modulating these interactions for the prevention of human diseases [93]. Major ‘omics’ approaches and emerging technologies already employed for nutritional research are overviewed in Table 1.

## 3. The Contribution of ‘Omics’ in Elucidating Rheumatoid Arthritis Pathogenesis

RA is an autoimmune condition that impacts predominantly the lining of the synovial joints and is characterized by chronic synovitis, systemic inflammation and various degrees of bone and cartilage erosion. Systemic inflammation characterizing RA is related with different extra-articular conditions, including cardiovascular diseases, resulting in higher mortality in patients with RA. In industrialized countries, RA is the most frequent form of inflammatory arthritis. The disorder is more common in women and the age of onset is typically between 25 and 50, in the midst of working life, with important social and economic burden [104]. Genetic factors can explain 50–60% of the risk [105,106] while environmental modifiable factors, such as infectious diseases, tobacco smoking, air pollution, dust, and nutrition, account for the remainder [107,108]. Thanks to the rapid progresses in the field of ‘omics’ technologies, the past decade has resulted in tremendous advancement in our capacity to interpret genetic and molecular reasons underlying complex conditions such as autoimmune diseases [109]. The etiopathology of RA is not fully understood, however, NGS combined with the application of innovative multi-omics approaches, cell profiling-technologies and bioinformatics tools has enabled a wider investigation and deeper insight into the pathogenesis and disease variants of RA, including the definition of RA-associated cell populations [110], specific gene expression profiles [111,112,113,114,115], susceptibility loci, gene-environment interactions, as well as genetic loci associated with subsets of patients and those linked with response to therapy and/or dietary components [116,117,118,119,120]. Genomics studies of RA, including recent application of genome wide association studies (GWAS), have discovered over 100 genetic loci linked with RA risk and/or severity [121]. Amid all the genetic susceptibility elements found so far, the human leukocyte antigen (HLA) locus is the more important one. A strong link between RA and *HLA-DRB1* alleles encoding an amino acid sequence pattern known as the ‘shared epitope’ has already been recognized for a long time. The shared epitope has been associated with a higher risk of anti-citrullinated protein antibodies (ACPAs)-positive RA while ACPAs positivity has been linked with more aggressive and destructive RA, systemic manifestations and cardiovascular complications [117]. It has been documented that the production of autoantibodies, including rheumatoid factor and ACPAs is an early and asymptomatic event that may precede for several years the onset of clinical apparent symptoms in RA. In the symptomatic RA phase, compensatory pathways that keep the disease asymptomatic may fail, promoting the transition from preclinical to clinically apparent disease [122]. These findings indicate an emerging disease paradigm where both genetic and environmental factors trigger a preclinical systemic autoimmune state, possibly originated at mucosal sites [123], followed by genetic and environmental factors that may propagate this silent autoimmune state to clinically overt RA [122,124].

Among the non-HLA gene variants identified there are many that involve immune system, citrullination, cytokine, and inflammation genes and/or known targets of approved therapy [121,125]. Loci showing stronger association with disease risk include genes encoding for protein tyrosine phosphatase, non-receptor type 22 (PTPN22), tumor necrosis factor (TNF) receptor-associated factor 1 (TRAF1), signal transducer and activator of transcription 4 (STAT4), interleukin 6 signal transducer (IL6ST), interleukin 2 receptor subunits alpha and beta, and CD40 [125]. The list of candidate genes is rapidly growing [126]. By combining the whole-genome sequencing and transcription profiles data of RA patients it is becoming possible to reveal the potential molecular pathways and crucial genes which play important roles in RA development as well as to identify the relevant cell types in RA pathogenesis [127,128,129]. Several pathways within the inflammatory cascade are activated in RA. All of them result in the upregulation of transcription factors and proinflammatory cytokines including nuclear factor kappa B (NFkB), interleukin (IL)-1, IL-6, IL-17, monocyte chemoattractant protein-1 (MCP-1) and tumor necrosis factor (TNF)- α. Diverse genes in RA loci are concerned with the NF-kB signaling pathway, the Janus kinase (JAK)-signal transducers and activators of transcription (STAT), or cytokines signaling pathways [130,131,132]. The release of cytokines promotes the activation of fibroblast-like synoviocytes (FLSs), macrophages, neutrophils, adhesion molecules, and clotting factors as well as the release of macrophage colony-stimulating factors, which increase macrophages and also induce osteoclast activation, promoting active bone destruction. Matrix metalloproteinases (MMPs), prostaglandins, cyclooxygenase-2 (COX-2) and free radicals are also produced in excessive amounts which enhance the inflammatory pathways, resulting in the breakdown of extracellular matrix and cartilage loss [133,134]. The local and systemic immune responses lead to the development of a persistent low-intensity inflammatory condition which is related to important RA comorbidities and complications, such as cardiovascular diseases, vascular damage, insulin-resistance, and amyloidosis [135,136,137,138]. Recent high-throughput and epigenome-wide analysis have allowed to demonstrate that epigenetics play vital roles in the pathogenesis of RA [49,139], providing a possible crossing point by which genetic and environmental risk factors interact each other to influence the susceptibility, onset, and development of RA. Indeed, DNA methylation, post-translational histone modifications and variations in gene expression induced by non-coding RNAs are implicated in adaptive and innate immune cell differentiation, migration, proliferation, apoptosis, and FLSs activation in RA patients [140,141]. Proteomics-based approaches have been utilized for the identification of key protein and peptide mediators in RA [142], as well as to detect and quantify cytokines, allowing the discovery of new potential biomarkers [143], which may serve for early diagnosis, as indicators for clinical observation on the disease progression and to monitor responses to therapeutic interventions. By studying biomarkers in patient populations, the disease could be sorted into different subcategories that show different outcomes and responses to specific drugs or nutritional interventions [120,144,145,146]. Multi-omics analysis integrated with advanced bioinformatic tools and machine learning are already yielding a more comprehensive understanding of molecular mechanisms underlying RA pathogenesis and therapeutic response, paving the way for personalized-medicine [147,148].

## 4. Nutrigenomics Approach to Rheumatoid Arthritis

It is already well documented from epidemiological studies that nutritional patterns could play a role both as disease risk and protective factor, on the basis of the properties of particular foods. Certain nutritional factors can indeed exhibit pro-inflammatory outcomes (for e.g., red meat, salt, excessive caloric intake) or conversely mitigate inflammation (for e.g., fruit and vegetables, flaxseeds, low caloric intake) [5,6]. Nutritional components derived from diet, such as glucids, amino acids, fatty acids, vitamins, minerals, and other natural compounds occurring in small quantities in food, can not only fulfil a structural role in the cell, but also represent molecular signals which may impact biochemical pathways and alter gene expression. Indeed, they can directly interrelate with key factors and regulate the pathways involved in inflammation cascades related to RA pathogenesis, or affect intra- and extracellular microenvironments, thereby indirectly altering cellular activities [149]. Christensen et al. have recently demonstrated the association between diet and immune cell-related gene expression patterns in humans [150]. Interestingly, reduction in pain and higher physical function in RA patients, as well as a lower risk of the disease in individuals carrying the HLA-DRB1 shared epitope allele, have been associated with adherence to the Mediterranean diet (MedDiet) [37,151]. The assigned health benefits of the MedDiet could be justified by a modulating effect on genes related to inflammation and oxidative stress [152]. Although nutrigenomic studies on RA patients are still scarce, the influence of several dietary patterns and bioactive compounds on inflammation-or other RA-related pathways has been investigated. For e.g., Camargo et al. (2013) showed that MedDiet is able to reduce the expression of NF-κB, TNF-α, MCP-1 and MMP-9 in human peripheral blood mononuclear cells [153]. Consumption of a MedDiet for 4 weeks has been associated with reduced IL-1β gene expression in human mononuclear cells in fasting and postprandial states [154]. It has also been shown that adherence to MedDiet is linked with changes of methylation-state in inflammation-related genes in peripheral blood cells [155]. In addition, recent studies have shown that the Mediterranean-based nutritional interventions are able to induce changes in the expression of inflammation-related miRNAs [100], possibly influencing RA risk by means of epigenetic mechanisms. MedDiet is characterized by frequent consumption of food from vegetable sources, whole grains, beans, nuts, seeds, legumes, fruit, and spices, while olive oil represents the principal source of fat. MedDiet also involves an equilibrate intake of fish and wine, as well as a lower consumption of red meat, sweets, and dairy products [156] Components of the MedDiet such as olive oil, vegetables, fruits, fatty-fish, and tree-nuts provide a model for functional foods, on the basis of their natural contents of bioactive compounds and nutraceuticals, such as flavonoids, alkaloids, polyphenols, terpenoids, sterols, pigments, and polyunsaturated fatty-acids of omega-3 series (omega-3 PUFAs). Olive oil holds a high nutritional value and a peculiar composition, which is particularly relevant in the case of Extra Virgin Olive Oil (EVOO). EVOO is considered an important bioactive food with several valuable properties and it may be efficacious in the management of inflammatory and autoimmune diseases, including RA [157,158]. It has been shown that the phenolic fraction of EVOO modulates cytokines production, including IL-6, TNF-α, and IL-1β, perhaps via NF-κB signaling pathway, exerting an anti-inflammatory and immunomodulatory action in systemic lupus erythematosus patients [159]. Castaner et al. (2012) have shown that three weeks ingestion of high-polyphenol-containing olive oil, reduce the activation of the CD40/CD40-ligand system and its downstream products in healthy subjects, as compared to olive oil containing less polyphenol [160]. About that, it would be worth noting that the inflammatory CD40/CD40-ligand pathway seems to be implicated in the progression from undifferentiated arthritis or ACPAs-positive arthralgia to established RA [161]. Moreover, a recent study has demonstrated that EVOO phenolic compounds may have a favorable effect on bone by modulating osteoblast-related gene expression, which would explain their beneficial effect against bone pathologies [162]. Omega-3 PUFAs could have a protective and preventive effect in RA, given their anti-inflammatory and pain-killer properties [163,164]. Epidemiological studies have reported a substantial inverse association between oily fish consumption and RA [165,166,167], suggesting that omega-3 PUFAs found in fish oil (eicosapentaenoic acid (EPA) and docosahexaenoic acid (DHA)) may be a protective factor against RA progression. In favour of this hypothesis, a case-control study established that RA cases had important decreased levels of EPA, and EPA+DHA in their erythrocyte membranes (a biomarker of omega-3 PUFAs status) compared with controls [168]. Moreover, Gan et al. (2017) showed that both omega-3 PUFAs supplement use and omega-3 PUFAs levels in red blood cell membranes were inversely linked with ACPAs positivity in people without RA, but at genetic risk for future RA [119]. A further study by the same authors found that the potential protective effect of omega-3 PUFAs on RA-related autoimmunity may be most pronounced in those who exhibit *HLA-DRB1* shared-epitope genetic variant [169]. Emerging evidences from epigenomic-wide association studies indicate that one of the mechanisms responsible for the omega-3 PUFAs-related anti-inflammatory modulation of gene expression within the cells involves the alteration of epigenetic markers, such as DNA methylation [97]. Inverse associations with risk of disease development and disease activity has been also associated with high intakes of fruit and vegetables, suggesting a protective factor [170,171]. Fruits, such as berries, pomegranates and citrus fruits, as well as vegetables and whole grains, are rich sources of a variety of vitamins and other bioactive compounds, especially the polyphenolic flavonoids that have been associated with anti-inflammatory, anti-oxidant and analgesic effects. Vegetarian and vegan dietary patterns are particularly high in such compounds and have been proven useful in the management of RA [172]. Moreover, several studies have demonstrated that patients with arthritis consume less fruits, whole grains and plant proteins, compared to those without arthritis [173,174,175]. Nutrigenomic effects of numerous dietary bioactive compounds naturally occurring in fruits, vegetables, and their derivative products, have been explored in humans. For e.g., resveratrol, a phenolic compound normally present in some fruits including red grapes, mulberries as well as in peanuts and red wine, has been shown to downregulate NF-κB, TNF-α, IL-β, IL-6 and COX-2 [176,177]. In addition, according to recent nutri-miromics studies, resveratrol is capable of downregulating miRNA-21, and miRNA-155 in different human cell culture models [98,99]. MiRNA-21 and miRNA-155 are highly expressed in RA, and are known to play critical roles in disease pathogenesis [178,179,180]. Moderate consumption of alcohol was negatively associated with the risk of developing ACPA-positive RA, especially in smokers carrying *HLA-DRB1* SE alleles [181], while excessive sodium chloride consumption among cigarette-smokers increases by more than two the risk of developing *HLA-DRB1* SE-positive RA, suggesting a significant additive effect between smoking, diet and genetic risk factors [182]. It has been shown that caffeine downregulates inflammatory pathways involved in autoimmunity. In vitro experiments showed significant downregulation at the mRNA levels of key inflammatory genes including STAT1 and TNF, and also cytokine levels were decreased significantly with caffeine treatment [183]. Another study has found that coffee decreases methotrexate (i.e., the first-line disease-modifying antirheumatic drug used to treat RA) intolerance and increases treatment compliance among RA patients [184]. Soukup et al. scrutinized the effect of coffee intake on the therapeutic impact of methotrexate in RA patients grouped based on different genotypes related to adenosine pathway. They found that genotypes and coffee consumption affect risk of RA and effectiveness of methotrexate treatment [118]. Based on genome-wide gene expression response data, Van Bussel et al. [185] have theorised that a brief term caloric restriction is effective in downregulating gene sets involved in the immune response in young men. Moreover, a recent multi-omics study has found that caloric restriction ameliorates insulin sensitivity in humans [186], while studies in human obesity and insulin resistance have shown a clear relationship between decreased insulin sensitivity, the chronic activation of pro-inflammatory signaling pathways and RA activity. Indeed, elevated levels of TNF-α and IL-6 have all been recorded in several diabetic and insulin-resistant conditions [187,188], while insulin-resistant pathways have been linked with disease activity in RA [189]. Various in vivo studies have stated the nutrigenomic impacts of single nutrient supplementations/withdrawal in mouse or rat models of RA, namely magnesium [190], methionine [191], nobiletin (a citrus flavone) [192], omega-3 PUFAs-derived metabolites [193], Equol (a major soybean phenolic metabolite) [194], epigallocatechin-3-gallate (a green tea polyphenol) [195] and numerous others, and all these interventions usually showed promise of favorably modulating some of the genes or pathways implicated in inflammation or arthritis in rodents. However, considering the differences between RA, as it occurs in humans, and the RA-like condition currently replicated in RA animal models [196], translatability of these in vivo studies to humans in order to predict the effects of bioactive compounds or nutritional interventions, may be questionable. Additional studies designed to identify the genetic susceptibility to RA and the interaction between particular genetic variants, RNAs and proteins expression, metabolites production and nutrient intake in humans, will be crucial in planning patient-specific nutritional interventions and preventive strategies based on patient genetic characteristics.

## 5. Rheumatoid Arthritis, Microbiome and Nutrition

It is well known that microbiome can affect the inflammatory state of an individual by influencing both the host innate and adaptive immune system and its metabolic potential [197]. In recent times, thanks also to the progress in microbiomics techniques which has allowed a deep characterization of microbial communities, the gut microbiota has been given an important role in RA aetiology and development [3,198,199].

Zhang et al. carried out metagenomic sequencing and a metagenome-wide association study on stool, salivary and dental specimens from a wide cohort of treatment-naïve RA patients and healthy controls. Consequently, they found that the gut microbiome and the oral microbiome displayed noteworthy dissimilarities between RA patients and control subjects, and, the altered gut microbiome and oral microbiome of RA patients were in part restored by DMARDs [200]. Other human microbiomics studies have revealed that RA patients display a decreased gut microbiota diversity if compared with healthy controls [201]. Patients with RA, especially erosive patients, have a characteristic enterotype of gut microbiota with a decreased abundance of bacteria belonging to the family *Bifidobacterium* and *Bacteroides* [202], an expansion of certain rare bacterial lineages [203], and, at least in the preclinic phase of the disease, an abundance of *Prevotella copri* in shared-epitope positive individuals [204]. It is now accepted that diet composition has a crucial role in the control of gut microbial populations and, thus, in the potential prevention, management and treatment of many human diseases, including RA [205]. During the past few years, numerous works have associated diet/nutrients, gut microbial communities and the expression of genes involved in immune responses [206]. Considering this relation, there may be important therapeutic benefit in manipulating microbial composition by dietary interventions. A fresh study by De Filippis et al. [88] has suggested that diet affect *P. copri* at the strain level. The authors analyzed the gut metagenomes of individuals with different dietary patterns, checking for the presence of distinct *P. copri* strains. They found that a diet high in fiber were linked to *P. copri* types with better ability in carbohydrate catabolism, while *P. copri* strains associated with an omnivore dietary pattern had a higher preponderance of genes involved in branched-chain amino acids (BCAA) biosynthesis. Whether particular strains are more prone to trigger RA than others, and whether different nutritional patterns could affect this risk, remains to be elucidated by further studies. However, it has been shown that high BCAA is a risk factor for type 2 diabetes, glucose intolerance, and promote pro-oxidant and pro-inflammatory activities in peripheral blood mononuclear cells [207], suggesting a possible relationship between *P. copri*, diet, and RA in genetically susceptible individuals, paving the way for precise nutritional interventions aiming to prevent RA. While the pathogenesis of microbiome-mediated diseases remains to be completely clarified, one mechanism may be linked to microbial metabolism. There is evidence that some foods have pro- or anti-inflammatory effects mediated by diet-related metabolites [208]. Metabolites generated by gut commensal microbes in response to host diet, may influence both microbiota and host homeostasis, with potentially beneficial or detrimental effects. Tang et al. have identified dietary compounds and phytochemicals that may affect microbiota abundance within the gut and interact with microbial community composition to change host metabolism. In particular long-term intake of plant-derived foods as well as consumption of artificial sweeteners were linked to important disparities in circulating metabolites, especially bile acids, which were linked on gut enterotype, suggesting that microbiome makeup and structure mediate the effect of nutrition on host physiology [91]. This could be interesting considering that bile acids are increasingly recognised as important signalling molecules in the regulation of immune homeostasis and inflammation [209]. Several metabolomics studies and genome-based analysis of bacteria have reported that the microbial metabolites regulate immune system and inflammation. For e.g., short chain fatty acids (SCFA), produced by bacteria that ferment fiber, are known to mediate immune functions and inflammation [210,211]. Moreover long-term vegetarian diet has been linked with an enrichment in butyrate-producing bacteria [212], while a short-term high-fiber nutritional intervention study in RA patients has been shown to increase anti-inflammatory SCFA and decrease pro-arthritic cytokines, along with a durable shift in the microbiome composition [102]. In addition, evidence suggests that SCFA may act as histone deacetylase inhibitors in human cells, thereby epigenetically modulating inflammation-related gene expression [213]. Trimethylamine N-oxide (TMAO), is an important microbiota-generated metabolite derived from dietary choline, betaine, and L-carnitine, which are contained in great amounts, in red meat, eggs, and dairy. TMAO is known to exert an impact on several significant mechanisms in the atherosclerosis pathogenesis pathway and vascular disfunction, promoting the etiological mechanisms of cardiovascular disease in RA [214]. Persistent red meat consumption increases systemic TMAO levels while high intake of meat and processed-meat products has been associated with an increased risk of RA [215,216], suggesting that this association could be mediated by the host-microbiome and microbial metabolome. Recently, it has been found that RA patients have a distinct oral microbiome. In addition, RA oral microbiota showed greater micobial diversity in comparison with healthy subjects, suggesting that there could be more potentially pathogenic microbes in the oral cavity of patients with RA and that this could negatively affect the outcome of the disease [217]. Tong et al. have observed a typical compositional modification of salivary microbiome in persons at increased risk for RA, indicating that oral microbiota dysbiosis arise in the preclinic stage of RA and are linked with systemic autoimmune features [218]. For e.g., it has been shown that *Porphyromonas gingivalis*, a bacterium which can be found the oral cavity, may generate an enzyme that citrullinates proteins. A close connection between the resulting inflammatory disorder of the oral mucosa (periodontitis) and a higher susceptibility to seropositive RA has been suggested [219]. Interestingly, some studies revealed that dietary habits can influence periodontal health, both directly, and indirectly by altering oral microbiota composition, [103,220,221], suggesting that some benefit might possibly be achieved through dietary modulation in at risk subjects.

## 6. Discussion

**Figure 1:**
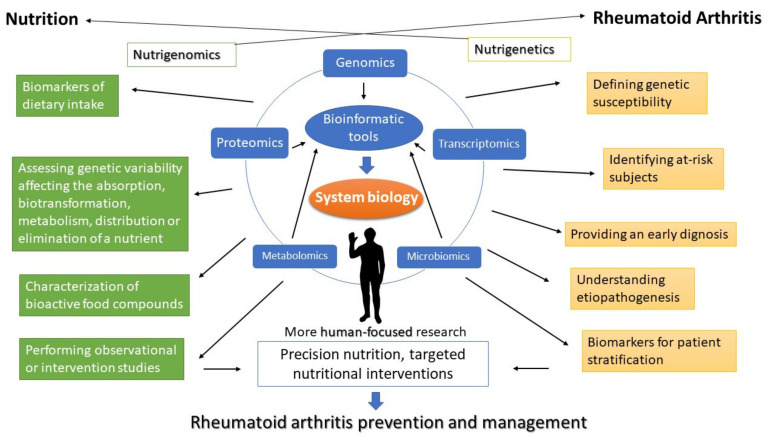
An integrative ‘omics’ approach will lead to a more human-focused research, precision nutrition, eventually resulting in better prevention strategies, as well as better management of rheumatoid arthritis.

The beginning of the 21st century was featured by fast progress in high-throughput ‘omics’ approaches, high-content-technologies, bioinformatics and computational power. Genomics, transcriptomics, proteomics, metabolomics, and microbiomics analysis now make it possible to improve the understanding of the pathogenesis of complex diseases such as RA as well as to study the relationship between nutrition, microbiota, health and disease at a molecular level. Thanks to the ‘omics’ methods and approaches, researchers are now experiencing the opportunity of connecting food components, diet, individual genetic background, health, and disease. Novel ‘omics’ technologies offer unprecedented opportunities to overcome the limitations inherent in traditional nutrition and RA research. Although robust associations between dietary intake and population health or disease are evident from conventional observational epidemiology, the outcomes of large-scale intervention studies examining the causality of those links have often proved unconvincing or have failed to demonstrate causality, including nutritional interventional studies related to RA [222,223]. This apparent conflict is most likely due to the well-known difficulty in assessing nutritional status and measuring habitual dietary intake which may lead to confounding in observational epidemiology. Indeed, dietary intake assessment is usually established by self-reported food intake questionnaires, which have intrinsic limitations. Metabolomics and proteomics benefit from the accessibility to the advanced high-sensitivity analytical tools to provide new opportunities for dietary biomarker development and application. Biomarkers of food or nutrient intake may allow to assess food consumption accurately and objectively by measuring blood/urine/fecal metabolites and avoiding the subjective errors that self-reporting of intake may introduce. There are several proteomics and metabolomics studies that have detected candidate biomarkers for diverse dietary habits, as well as for several kinds of foods, including meat, vegetables and fruits. Numerous studies have also described metabolites associated to specific dietary patterns, like MedDiet, high fat, or Western diets [208]. Interestingly, some studies revealed increased levels of carnitine and taurine in patients with active RA [224,225]. Carnitine and taurine are known to be potential biomarkers of meat intake thus this might support the evidences from epidemiological studies which correlate high meat consumption with RA [215]. The identification of food biomarkers is an ongoing process. The application of biomarkers in nutritional research will be crucial to ameliorate the assessment of dietary intake, exposure to particular nutritional components, and of compliance to nutritional interventions, as well providing information on interindividual differences in response to diet. There is a large need for further studies to better understand the influence of diet or dietary bioactive components on RA risk, possibly encompassing a nutrigenomics approach. This approach will lead to precision nutrition, whose goals are the prevention and management of chronic diseases, such as RA, by adjusting nutritional interventions or recommendations according to a specific genetic background or metabolic profile. Even if dietary biomarkers usually allow for a more realistic measure of nutritional intake, some factors which are absent in the conventional methods of dietary assessment could bias the measurement of dietary intake biomarkers. Such factors include genetic variations, nutritional factors (e.g., nutrient-nutrient interaction), lifestyle/physiological conditions (e.g., smoking), biological sampling and analytical methodology. However, existing research on this issue is still scarce. Currently there is no agreement as to which metabolites would be the most suitable biomarkers for distinct kind of foods. By the way, some metabolites are indicators of groups of food, not being capable to distinguish between the exact kinds of foods being examined (for e.g., a metabolite typically found in meat may not be useful in discerning between different types of meat). Possibly, for several foods, an association of diverse metabolites would be more suitable as a marker than a single metabolite. Unfortunately, the multi-omics studies in RA have not gathered dietary intake data nor employed the same ‘omics’ platforms so far, hampering the connection of specific food intake with metabolic changes in RA patients. Thus it is essential to evaluate a biomarker’s reliability, reproducibility, ability to reveal modifications over time and robustness throughout different populations, as well as advantages and disadvantages to warrant it is assessed using the adequate techniques [208,226]. Since conducting nutrition research with human subjects may be often challenging, many studies aimed to explore the mutual relationship between autoimmune diseases, including RA, nutrition and microbiota have been- and are being carried out in animal models. However, while animal models have been helpful in clarifying the fundamental mechanisms underpinning RA pathogenesis and immune responses, the use of non-human models to replicate the complexity of the relationship between RA, diet, microbiota as well as to evaluate the effects of nutritional interventions, may possibly be misleading, taking into account the several interspecies differences characterizing for e.g., physiologic responses to nutrients and systemic inflammatory challenges [227,228], gastrointestinal physiology and microbiome composition [229]. Recent developments in stem cell biology and three-dimensional complex fluidic in vitro systems offer exciting opportunities for developing new human biology-based models for use in nutrigenomics, microbiota and RA omics-based research [198,229]. Such emerging tools, which are already being applied in biomedical research, are likely to be better models of the complexity of the human in vivo situation. The integration of these tools with nutrigenomics and microbiomics could provide nutrition researchers with huge opportunities to undertake well-controlled experiments using very manageable human-relevant models to investigate the mechanisms through which food components modulate gene and protein expression, metabolite production and epigenetic pathways in both health and disease. Individually, ‘omics’ technologies have promoted a critical shift in biomedical and nutrition sciences. Anyhow, each approach individually cannot grab the entire biological complexity of nutrition-disease relationships. Combination of multiple technologies, referred to as ‘multi-omics approach or systems biology’ is emerging as an approach to allow a broader view of biology, disease and the influences of environmental factors, including diet [230]. The integration of data from different ‘omics’ platforms (system biology) could provide multidimensional insight into the relationship between pathogenetic processes and the influence of nutrition, allowing the retrieval of comprehensive and holistic biological information. Although an “inflammatory” dietary pattern may have a role in the switch from preclinical to clinical RA, and early nutritional intervention might possibly result in a delay or prevention of the onset of RA, it requires both an early diagnosis and the identification of at risk population [174,231]. Since current diagnostic tests are not sufficiently sensitive or accurate in the very early stages of the disease, RA is typically diagnosed only once damage to the joints has already begun, a time at which the window for optimal interventions may have been missed. The multi-omics approach has the potential to identify multiple biomarkers that can be used to revolutionize the management of RA by mean of enabling a timely diagnosis. Furthermore, through the analysis of biomarkers in patient populations, the disease could be stratified into distinct subsets that exhibit differential risks, outcomes, and eventually different responses to specific foods or dietary interventions. The integration of diverse complex ‘omics’ datasets may be considered one of the key challenges of today’s bioinformatics, due to different data formats, high data dimensionality and need for data normalization [232,233]. The constant exponential growth in ‘omics’ data requires a parallel development in computing power and software systems for handling this challenge. New bioinformatic stuffs for the integration of data from several ‘omics’ fields continue to arise, and will assist researchers to faithfully decode data in the context of biological systems, but harmonized actions are essential to encourage this process [234]. The multi-omics approach will provide the broader scientific community with a valuable resource to address many questions about RA pathogenesis and disease-nutrition interactions at a system-biology level, resulting in development of new diagnostic tools, therapeutic strategies, and targeted nutritional interventions, resulting in better disease prevention and management (Figure 1).

## 7. Conclusions

The continuous advancement in ‘omics’ technologies has a huge potential to transform nutrition and RA research. The application and integration of novel ‘omics’ technologies together with advanced computational tools will enable a better understanding of RA pathogenetic mechanisms at a molecular level, the discovery of new biomarkers, the identification and characterization of food bioactive compounds and their impact on RA-related pathways. This will lead to a more human-relevant approach to nutrition- and RA- research that ultimately will lead to a nutrigenomics approach and precision nutrition, for future better disease prevention and management.

## Figures and Tables

**Table 1:** Examples of ‘omics’ approaches and emerging technologies employed in nutritional research. Five major categories and their subcategories are shown.

Approach	Subcategory	Targets	Techniques	References
**Genomics**	Genomics	Genes (DNA sequence)	Second generation: Illumina; SOLID; Ion Torrent. Third generation: PacBio; SMRT-seq; Illumina Tru-seq Synthetic Long-Read technology; Oxford Nanopore Technologies sequencing platform	[65,94]
	Epigenomics	Modification of DNA and DNA- binding proteins	Whole-genome bisulfite sequencing (for DNA Methylation Analysis); ChIP-seq (for DNA–Protein Interaction Analysis); ATAC-Seq (for Chromatin Accessibility Analysis)	[95,96,97]
**Transcriptomics**	Transcriptomics	mRNA	RNA-microarrays; RNA-seq: Illumina, SOLID, Ion Torrent (second generation); PacBio, SMRT-seq, Illumina TruSeq Synthetic Long-Read technology, Oxford Nanopore Technologies sequencing platform (third generation)	[47,48,49,50]
	ncRNA-omics	non-coding RNA (including microRNA)		[57,98,99,100]
**Proteomics**	Proteomics	Proteins	Protein-microarrays; NMR Spectroscopy; MS; single-cell and ultrasensitive protein analyses	[79,80,81]
	Interactomics	Protein-protein interaction, protein-small molecules interaction	TAP; Affinity Chromatography; Coimmunoprecipitation; Protein arrays; PFC; Phage display, NMR spectroscopy	[84,85,101]
**Metabolomics**	Metabolomics	Metabolites	NMR; ^1^H NMR; MS; MALDI-TOF; SIMS; FTICR-MS	[72,73,74,75,76,77,78]
	Lipidomics	Lipids		
	Aminomics	Aminoacids		
**Microbiomics**	Microbiomics	Human Microbiota (including bacteria, fungi, protozoa, and viruses)		[87,88,89,90,91,102,103]
	Meta-genomics	Microbiota DNA		
	Meta-transcriptomics	Microbiota RNAs		
	Meta-proteomics	Microbiota proteins		
	Meta-bolomics	Microbiota metabolites		

Abbreviations: ATAC-Seq, Assay for transposase-accessible chromatin-Squencing; ChIP-seq, Chromatin immunoprecipitation-Sequencing; FTICR-MS, Fourier transform ion cyclotron resonance-Mass Spectrometry. ^1^H NMR, Proton Nuclear Magnetic Resonance spectroscopy; lncRNAs, long non-coding RNAs; MALDI-TOF, Matrix-Assisted Laser Desorption Ionization Time of Flight; MS, Mass Spectrometry; NMR, Nuclear Magnetic Resonance; PacBio, Pacific Biosciences; PFC, Protein fragment Complementation; SIMS, Secondary Ion Mass Spectrometry; SMRT-seq, Single Molecule Real Time sequencing; SOLID: Sequencing by Oligonucleotide Ligation and Detection; TAP, Tandem Affinity Purification.

## Data Availability

Data sharing is not applicable to this article. No new data were created in this study.

## Supplementary Materials

The following are available online at https://www.mdpi.com/2072-6643/13/3/763/s1, Table S1: Supplementary information about applied search terms in PubMed.
