# Supplementary material for: Nutrition and Rheumatoid Arthritis in the ‘Omics’ Era
Source: Nutrients. 2021 Feb 26;13(3):763. doi: 10.3390/nu13030763 (PMC7996781; doi:10.3390/nu13030763)
Supplement: Supplementary file 1 [file nutrients-13-00763-s001.pdf]

Table S1: Supplementary information about applied search terms in PubMed

Search was conducted using PubMed (in August-November 2020) applying the list of keywords (with MeSh terms) and Boolean operators indicated in the table below. Only human-based studies (e.g., observational and interventional studies in RA and inflammation) and papers written in English were reviewed.

Topic	Keywords combinations
Omics and nutrition	(("nutritional status"[MeSH Terms] OR "nutritional sciences"[MeSH Terms] OR ("nutrition s"[All Fields] OR "nutritional status"[MeSH Terms] OR ("nutritional"[All Fields] AND "status"[All Fields]) OR "nutritional status"[All Fields] OR "nutrition"[All Fields] OR "nutritional sciences"[MeSH Terms] OR ("nutritional"[All Fields] AND "sciences"[All Fields]) OR "nutritional sciences"[All Fields] OR "nutritional"[All Fields] OR "nutritionals"[All Fields] OR "nutritions"[All Fields] OR "nutritive"[All Fields])) AND ("genome"[MeSH Terms] OR "genome"[All Fields] OR "genomes"[All Fields] OR "genome s"[All Fields] OR "genomically"[All Fields] OR "genomics"[MeSH Terms] OR "genomics"[All Fields] OR "genomic"[All Fields])) OR "transcrpitomics"[All Fields] OR ("proteom"[All Fields] OR "proteome"[MeSH Terms] OR "proteome"[All Fields] OR "proteomes"[All Fields] OR "proteomical"[All Fields] OR "proteomically"[All Fields] OR "proteomics"[MeSH Terms] OR "proteomics"[All Fields] OR "proteomic"[All Fields]) OR ("metabolome"[MeSH Terms] OR "metabolome"[All Fields] OR "metabolomes"[All Fields] OR "metabolomics"[MeSH Terms] OR "metabolomics"[All Fields] OR "metabolomic"[All Fields])<br><br>((((((((((((("nutrition research") AND (genomics)) OR (genomics[MeSH Terms])) OR (transcriptomics[MeSH Terms])) OR (transcriptomics)) OR (proteomics)) OR (proteomics[MeSH Terms])) OR (metabolomics[MeSH Terms])) OR (metabolomics)) OR (epigenetics))) OR (epigenetics[MeSH Terms])) OR (miRNA[MeSH Terms])) OR (miRNAs)<br><br>("nutrition s"[All Fields] OR "nutritional status"[MeSH Terms] OR ("nutritional"[All Fields] AND "status"[All Fields]) OR "nutritional status"[All Fields] OR "nutrition"[All Fields] OR "nutritional sciences"[MeSH Terms] OR ("nutritional"[All Fields] AND "sciences"[All Fields]) OR "nutritional sciences"[All Fields] OR "nutritional"[All Fields] OR "nutritionals"[All Fields] OR "nutritions"[All Fields] OR "nutritive"[All Fields] OR ("nutritional status"[MeSH Terms] OR "nutritional sciences"[MeSH Terms])) AND "next generation sequencing"[All Fields]<br><br>(("diet"[MeSH Terms] OR ("diet"[MeSH Terms] OR "diet"[All Fields]) OR ("nutrition s"[All Fields] OR "nutritional status"[MeSH Terms] OR ("nutritional"[All Fields] AND "status"[All Fields]) OR "nutritional status"[All Fields] OR "nutrition"[All Fields] OR "nutritional sciences"[MeSH Terms] OR ("nutritional"[All Fields]

	AND "sciences"[All Fields]) OR "nutritional sciences"[All Fields] OR "nutritional"[All Fields] OR "nutritionals"[All Fields] OR "nutritions"[All Fields] OR "nutritive"[All Fields])) AND ("transcriptom"[All Fields] OR "transcriptomal"[All Fields] OR "transcriptome"[MeSH Terms] OR "transcriptome"[All Fields] OR "transcriptomes"[All Fields] OR "transcriptomic"[All Fields] OR "transcriptomically"[All Fields] OR "transcriptomics"[All Fields])) OR ("sequence analysis, rna"[MeSH Terms] OR ("sequence"[All Fields] AND "analysis"[All Fields] AND "rna"[All Fields]) OR "rna sequence analysis"[All Fields] OR ("rna"[All Fields] AND "sequencing"[All Fields]) OR "rna sequencing"[All Fields]) OR "sequence analysis, rna"[MeSH Terms]
Omics and Rheumatoid arthritis	(((rheumatoid arthritis) OR (rheumatoid arthritis[MeSH Terms])) AND (next generation sequencing)) OR (next generation sequencing[MeSH Terms])<br><br>(("arthritis, rheumatoid"[MeSH Terms] OR ("arthritis"[All Fields] AND "rheumatoid"[All Fields]) OR "rheumatoid arthritis"[All Fields] OR ("rheumatoid"[All Fields] AND "arthritis"[All Fields])) AND ("genome"[MeSH Terms] OR "genome"[All Fields] OR "genomes"[All Fields] OR "genome s"[All Fields] OR "genomically"[All Fields] OR "genomics"[MeSH Terms] OR "genomics"[All Fields] OR "genomic"[All Fields])) OR "genomics"[MeSH Terms] OR ("transcriptom"[All Fields] OR "transcriptomal"[All Fields] OR "transcriptome"[MeSH Terms] OR "transcriptome"[All Fields] OR "transcriptomes"[All Fields] OR "transcriptomic"[All Fields] OR "transcriptomically"[All Fields] OR "transcriptomics"[All Fields]) OR ("proteom"[All Fields] OR "proteome"[MeSH Terms] OR "proteome"[All Fields] OR "proteomes"[All Fields] OR "proteomical"[All Fields] OR "proteomically"[All Fields] OR "proteomics"[MeSH Terms] OR "proteomics"[All Fields] OR "proteomic"[All Fields]) OR "proteomics"[MeSH Terms] OR "metabolomics"[MeSH Terms] OR ("metabolome"[MeSH Terms] OR "metabolome"[All Fields] OR "metabolomes"[All Fields] OR "metabolomics"[MeSH Terms] OR "metabolomics"[All Fields] OR "metabolomic"[All Fields]) OR ("epigenetical"[All Fields] OR "epigenetically"[All Fields] OR "epigenomics"[MeSH Terms] OR "epigenomics"[All Fields] OR "epigenetic"[All Fields] OR "epigenetics"[All Fields]) OR "epigenomics"[MeSH Terms] OR "micrornas"[MeSH Terms] OR ("micrornas"[MeSH Terms] OR "micrornas"[All Fields] OR "mirna"[All Fields] OR "mirnas"[All Fields] OR "mirna s"[All Fields])<br><br>(((((((microbiomics) OR (microbiota[MeSH Terms])) OR (microbiota)) OR (microbiome[MeSH Terms])) OR (microbiome)) AND (rheumatoid arthritis)) OR (rheumatoid arthritis[MeSH Terms])) OR (inflammation)<br><br>(((rheumatoid arthritis) OR (rheumatoid arthritis[MeSH Terms])) AND (pathogenesis)) AND ("system biology")

Rheumatoid arthritis and nutrigenomics	(("arthritis, rheumatoid"[MeSH Terms] OR ("arthritis"[All Fields] AND "rheumatoid"[All Fields]) OR "rheumatoid arthritis"[All Fields] OR ("rheumatoid"[All Fields] AND "arthritis"[All Fields]) OR "arthritis, rheumatoid"[MeSH Terms]) AND ("nutrigenomics"[MeSH Terms] OR "nutrigenomics"[All Fields] OR "nutrigenomic"[All Fields])) OR "nutrigenomics"[MeSH Terms] OR "nutritional genomics"[All Fields]<br><br>("arthritis, rheumatoid"[MeSH Terms] OR ("arthritis"[All Fields] AND "rheumatoid"[All Fields]) OR "rheumatoid arthritis"[All Fields] OR ("rheumatoid"[All Fields] AND "arthritis"[All Fields]) OR "arthritis, rheumatoid"[MeSH Terms] OR ("inflammation"[MeSH Terms] OR "inflammation"[All Fields] OR "inflammations"[All Fields] OR "inflammation s"[All Fields]) OR "inflammation"[MeSH Terms] OR "oxidative stress"[MeSH Terms] OR "oxidative stress"[All Fields]) AND "extra virgin olive oil"[All Fields] AND ("gene expression"[MeSH Terms] OR ("gene"[All Fields] AND "expression"[All Fields]) OR "gene expression"[All Fields]) AND "gene expression"[MeSH Terms]<br><br>((((((("arthritis, rheumatoid"[MeSH Terms] OR ("arthritis"[All Fields] AND "rheumatoid"[All Fields]) OR "rheumatoid arthritis"[All Fields] OR ("rheumatoid"[All Fields] AND "arthritis"[All Fields]) OR "arthritis, rheumatoid"[MeSH Terms] OR ("inflammation"[MeSH Terms] OR "inflammation"[All Fields] OR "inflammations"[All Fields] OR "inflammation s"[All Fields]) OR "inflammation"[MeSH Terms] OR "oxidative stress"[MeSH Terms] OR "oxidative stress"[All Fields])) AND (omega 3[MeSH Terms])) OR ("Eicosapentaenoic acid")) OR ("docosahexaenoic acid")) OR (acids, docosahexaenoic[MeSH Terms])) OR (acids, unsaturated fatty[MeSH Terms])) OR (n 3 pufa[MeSH Terms])<br><br>(((((((("arthritis, rheumatoid"[MeSH Terms] OR ("arthritis"[All Fields] AND "rheumatoid"[All Fields]) OR "rheumatoid arthritis"[All Fields] OR ("rheumatoid"[All Fields] AND "arthritis"[All Fields]) OR "arthritis, rheumatoid"[MeSH Terms] OR ("inflammation"[MeSH Terms] OR "inflammation"[All Fields] OR "inflammations"[All Fields] OR "inflammation s"[All Fields]) OR "inflammation"[MeSH Terms] OR "oxidative stress"[MeSH Terms] OR "oxidative stress"[All Fields])) AND (gene expression[MeSH Terms])) OR ("gene expression")) OR ("epigenetic")))) AND (nutrition[MeSH Terms])) OR (dietary habit[MeSH Terms])<br><br>(("nutrigenetic"[All Fields] OR "nutrigenomics"[MeSH Terms] OR "nutrigenomics"[All Fields] OR "nutrigenetics"[All Fields] OR ("nutrigenomics"[MeSH Terms] OR "nutrigenomics"[All Fields] OR "nutrigenomic"[All Fields]) OR "nutrigenomics"[MeSH Terms] OR "nutrigenomics"[MeSH Terms]) AND "arthritis, rheumatoid"[MeSH Terms]) OR ("arthritis, rheumatoid"[MeSH Terms] OR ("arthritis"[All Fields] AND "rheumatoid"[All Fields]) OR "rheumatoid arthritis"[All Fields] OR ("rheumatoid"[All Fields] AND "arthritis"[All Fields]))

Rheumatoid arthritis, nutrition and omics	("arthritis, rheumatoid"[MeSH Terms] OR ("arthritis"[All Fields] AND "rheumatoid"[All Fields]) OR "rheumatoid arthritis"[All Fields] OR ("rheumatoid"[All Fields] AND "arthritis"[All Fields])) AND ("nutrition s"[All Fields] OR "nutritional status"[MeSH Terms] OR ("nutritional"[All Fields] AND "status"[All Fields]) OR "nutritional status"[All Fields] OR "nutrition"[All Fields] OR "nutritional sciences"[MeSH Terms] OR ("nutritional"[All Fields] AND "sciences"[All Fields]) OR "nutritional sciences"[All Fields] OR "nutritional"[All Fields] OR "nutritionals"[All Fields] OR "nutritions"[All Fields] OR "nutritive"[All Fields]) AND ("omics"[Journal] OR "omics"[All Fields])<br><br>(rheumatoid arthritis) AND **(**(nutrition) OR (omics)**)**<br>allora la stringa che ti dà è cosi:<br>("arthritis, rheumatoid"[MeSH Terms] OR ("arthritis"[All Fields] AND "rheumatoid"[All Fields]) OR "rheumatoid arthritis"[All Fields] OR ("rheumatoid"[All Fields] AND "arthritis"[All Fields])) AND ("nutrition s"[All Fields] OR "nutritional status"[MeSH Terms] OR ("nutritional"[All Fields] AND "status"[All Fields]) OR "nutritional status"[All Fields] OR "nutrition"[All Fields] OR "nutritional sciences"[MeSH Terms] OR ("nutritional"[All Fields] AND "sciences"[All Fields]) OR "nutritional sciences"[All Fields] OR "nutritional"[All Fields] OR "nutritionals"[All Fields] OR "nutritions"[All Fields] OR "nutritive"[All Fields] OR ("omics"[Journal] OR "omics"[All Fields]))
Microbiomics, nutrition and rheumatoid arthritis	(((((((((microbiomics) OR (microbiota[MeSH Terms])) OR (microbiota)) OR (microbiome[MeSH Terms])) OR (microbiome)) AND (rheumatoid arthritis)) OR (rheumatoid arthritis[MeSH Terms])) OR (inflammation)) AND (nutrition)) OR (nutrition[MeSH Terms])) OR ("diet")<br><br>(((((((((microbiomics) OR (microbiota[MeSH Terms])) OR (microbiota)) OR (microbiome[MeSH Terms])) OR (microbiome)) AND (rheumatoid arthritis)) OR (rheumatoid arthritis[MeSH Terms])) OR (inflammation))) AND ("next generation sequencing")) OR ("long read sequencing")<br><br>((("metagenomics"[MeSH Terms] OR ("metagenome"[MeSH Terms] OR "metagenome"[All Fields] OR "metagenomes"[All Fields] OR "metagenomic"[All Fields] OR "metagenomically"[All Fields] OR "metagenomics"[MeSH Terms] OR "metagenomics"[All Fields])) AND ("microbiota"[MeSH Terms] OR "microbiota"[All Fields] OR "microbiotas"[All Fields] OR "microbiota s"[All Fields] OR "microbiotae"[All Fields])) OR "microbiota"[MeSH Terms]) AND ("arthritis, rheumatoid"[MeSH Terms] OR ("arthritis"[All Fields] AND "rheumatoid"[All Fields]) OR "rheumatoid arthritis"[All Fields] OR ("rheumatoid"[All Fields] AND "arthritis"[All Fields]))
